# Evaluation of Ultra-Low-Dose CBCT Protocols to Investigate Vestibular Bone Defects in the Context of Immediate Implant Planning: An Ex Vivo Study on Cadaver Skulls

**DOI:** 10.3390/jcm14124196

**Published:** 2025-06-12

**Authors:** Mats Wernfried Heinrich Böse, Jonas Buchholz, Florian Beuer, Stefano Pieralli, Axel Bumann

**Affiliations:** 1 Department of Prosthodontics, Geriatric Dentistry and Craniomandibular Disorders, Charité—Universitätsmedizin Berlin, Corporate Member of Freie Universität Berlin and Humboldt-Universität zu Berlin, Aßmannshauser Str. 4-6, 14197 Berlin, Germany; jonas.buchholz@charite.de (J.B.); florian.beuer@charite.de (F.B.); stefano.pieralli@charite.de (S.P.); 2 A+ Orthodontic, Georgenstraße 25, 10117 Berlin, Germany; ab@aplus-kieferorthopaeden.de

**Keywords:** cone-beam computed tomography, alveolar bone loss, diagnostic imaging, dental implants, digital technology, intraoral scan, digital workflow

## Abstract

**Background/Objectives:** This ex vivo study aimed to evaluate the diagnostic performance of ultra-low-dose (ULD) cone-beam computed tomography (CBCT) protocols in detecting vestibular bone defects for immediate implant planning, using intraoral scan (IOS) data as a reference. **Methods:** Four CBCT protocols (ENDO, A, B, C) were applied to four dried human skulls using a standardized setup and a single CBCT unit (Planmeca ProMax^®^ 3D Mid, Planmeca Oy, Helsinki, Finland). All scans were taken at 90 kV, with varying parameters: (1) ENDO (40 × 50 mm, 75 µm, 12 mA, 80–120 µSv, 15 s), (2) A (50 × 50 mm, 75 µm, 9 mA, 20–40 µSv, 5 s), (3) B (100 × 60 mm, 150 µm, 7.1 mA, 22–32 µSv, 5 s), and (4) C (100 × 100 mm, 200 µm, 7.1 mA, 44 µSv, 4 s). Vestibular root surfaces of single-rooted teeth (FDI regions 15–25 and 35–45) were digitized via IOS and exported as STL files. CBCT datasets were superimposed using 3D software (Blender 2.79), and surface defects were measured and compared using one-sample *t*-tests and Bland–Altman analysis. The level of significance was set at *p* < 0.05. **Results:** A total of 330 vestibular surfaces from 66 teeth were analyzed. Compared to the IOS reference, protocols ENDO and A showed minimal differences (*p* > 0.05). In contrast, protocols B and C exhibited statistically significant deviations (*p* < 0.05). Protocol B demonstrated a mean difference of −0.477 mm^2^ with limits of agreement (LoA) from −2.04 to 1.09 mm^2^ and significant intra-rater variability (*p* < 0.05). Protocol C revealed a similar mean deviation (−0.455 mm^2^) but a wider LoA (−2.72 to 1.81 mm^2^), indicating greater measurement variability. Overall, larger voxel sizes were associated with increased random error, although deviations remained within clinically acceptable limits. **Conclusions:** Despite statistical significance, deviations for protocols B and C remained within clinically acceptable limits. ULD CBCT protocols are, thus, suitable for evaluating vestibular bone defects with reduced radiation exposure.

## 1. Introduction

Immediate implant placements seem to offer advantages regarding treatment time, patient comfort, and the preservation of hard and soft tissues [1,2]. Thereby, comparable results to delayed implant protocols were documented in the literature after a short 1-year observation period [3,4,5,6]. However, remodeling processes after tooth extraction and immediate implant installation are not completely preventable [7,8,9], increasing the risk of technical, biological, and esthetic complications. Therefore, bone availability and the extent of possible defects should be accurately determined before surgery [10,11,12], as it might affect long-term clinical outcomes [13,14,15].

To obtain reliable three-dimensional (3D) radiological data while reducing radiation exposure, cone-beam computed tomography (CBCT) has been established within digital implant planning workflows [16,17,18]. It thereby offers more important and helpful information regarding the existing hard and soft tissues compared to conventional panoramic X-rays [16,19,20,21]. For the acquisition of CBCT scans, voxel sizes of 0.3 to 0.4 mm are typically recommended, while some authors consider voxel sizes of 0.15 to 0.2 mm to be superior for visualizing thin vestibular bone lamellae and associated defects [11,22,23,24,25]. In recent years, low-dose (LD) and ultra-low-dose (ULD) CBCT protocols have continuously been developed and improved. While they lead to substantially lower effective doses by modifying voxel size, voltage, current intensity, or exposure time [26,27,28], they are still viewed critically by practitioners due to their lower image quality.

Nevertheless, in 2017, the ALARA (As Low As Reasonably Achievable) and ALADA (As Low As Diagnostically Acceptable) principles [29] were advanced by DIMITRA (Dentomaxillofacial Pediatric Imaging: an Investigation Toward Low Dose Radiation-Induced Risks), a European research group [30]. With a focus on enhancing radiation protection, ALADAIP (As Low As Diagnostically Acceptable, being indication-oriented and patient-specific) was introduced, shifting the emphasis from obtaining the “best images” to acquiring indication-oriented images while minimizing dose exposure [31]. This approach reflects the varying requirements for image usability and aims to balance image quality with effective dose, thereby reinforcing the justification for LD and ULD CBCT protocols. Different studies evaluated sectional views of CBCTs in which transversal and vertical bone defects were shown as accurately as possible [23,32,33]. Thereby, one-dimensional (1D) information was extracted from 3D datasets of CBCTs [34,35]. While offering highly accurate and reproducible measurements with this procedure [22,23], reliable evaluations of complete alveolar bone defects should include an analysis of the whole area. One-dimensional measures do not allow for conclusions to be made regarding the complete defect size. Some authors have categorized these differences, but no universally accepted standard has yet been developed [14,36].

Therefore, the present study aimed to evaluate the diagnostic performance of ULD CBCT protocols in detecting vestibular bone defects for immediate implant planning, using IOS data as a reference. The hypothesis was that the measurements of bony defects with different ULD protocols do not differ from those measured with an IOS serving as a reference.

## 2. Materials and Methods

The present study was designed as an ex vivo study on cadaver skulls. Ethical approval was given by the local ethics committee (application number: EA2/160/18). CBCTs (Planmeca ProMax^®^ 3D Mid, Planmeca Oy, Helsinki, Finland) of four dried skulls with mostly undamaged maxilla and mandible from the anatomical collection of a local Specialty Network Anatomy were taken. Subsequently, measured vestibular bone defects were compared with those obtained from an IOS, which served as reference datasets. The entire experimental series was conducted by a single operator (author J.B.) under the supervision of the author A.B.

The following protocols were used when preparing CBCTs, as they represent regularly used settings:

- The protocol with the highest resolution (ENDO);
- The corresponding ULD protocol (same FOV, voltage, and voxel size; protocol A);
- A high-resolution protocol of the maxilla and mandible with a voxel size of 150 µm (protocol B);
- A high-resolution protocol with a larger FOV and a voxel size of 200 µm (protocol C).

Detailed settings are shown in Table 1.

### 2.1. Experimental Setup

Initially, vestibular exposed root surfaces of all suitable teeth—specifically Fédération Dentaire Internationale (FDI) regions 15 to 25 and 35 to 45, which are single-rooted with a clearly identifiable cementoenamel junction—along with the adjacent structures of all skulls, were scanned using an intraoral scanner (CS 3600, Carestream Dental LLC, Atlanta, GA, USA, IOS). The scan data were subsequently exported from the IOS software as Standard Triangulation Language (STL) files to serve as references for further analysis.

Subsequently, CBCT scans were acquired using different settings based on the protocols outlined in Table 1. Due to the partially limited field of view (FOV), separate CBCT scans of jaws and regions were sometimes required to fully capture the dental arches in FDI region 15–25. Consequently, and resulting from manufacturer-specific settings regarding different regions and jaws, a dose range is specified for protocols ENDO, A, and B. To simulate an upright patient position during image acquisition, all skulls were stabilized using foam supports. The resulting scans were processed using the proprietary software provided by the manufacturer (Planmeca Romexis 5.1.1.R, Planmeca Oy, Helsinki, Finland), which offers various tools for 2D and 3D image editing. For this study, only the 3D functionality was necessary. Each scan was rendered and exported as an STL file. For this process, the default 3D rendering settings for hard tissues were applied, including contrast (1080), brightness (1580), density (928), and translucency (0). The software converts the segmented voxel data from the CBCT scan into a triangulated mesh surface, enabling STL file generation and comparison with IOS-generated data.

**Figure 1:**
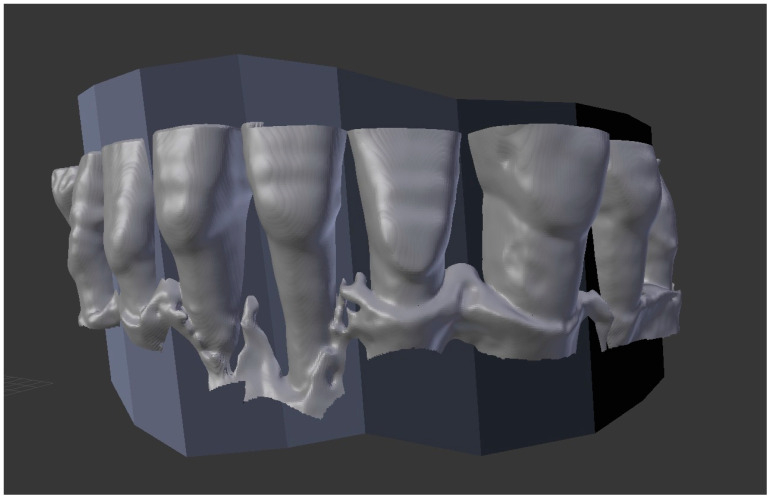
Exemplary rendering, including the segmentation surface using the 3D graphic software when displaying an STL dataset from the ENDO protocol.

Further post-processing was carried out after importing respective STL files into an open-source 3D graphics software (Blender 2.79, Blender Foundation, Amsterdam, The Netherlands). All STL datasets were individually reduced to structures relevant to the implementation of this study by marking and deleting unneeded vertices (i.e., excluded molars, ascending ramus, or other excluded anatomical structures). Additionally, a consistent segmentation surface was necessary. It was individually created using the STL files of all obtained intraoral scans (reference datasets), following the incisal edge from mesial to distal of respective teeth (Figure 1). It was constructed to form a straight and consistent surface regarding each tooth to provide a clear segmentation for vestibular surface evaluation of all obtained datasets. Through a combination of manual fitting and an Iterative Closest Point (ICP) tool, all imported STL files from CBCT scans could be superimposed to respective segmentation surfaces of reference datasets, enabling an independent evaluation of vestibular bone defects (Figure 1).

**Figure 2:**
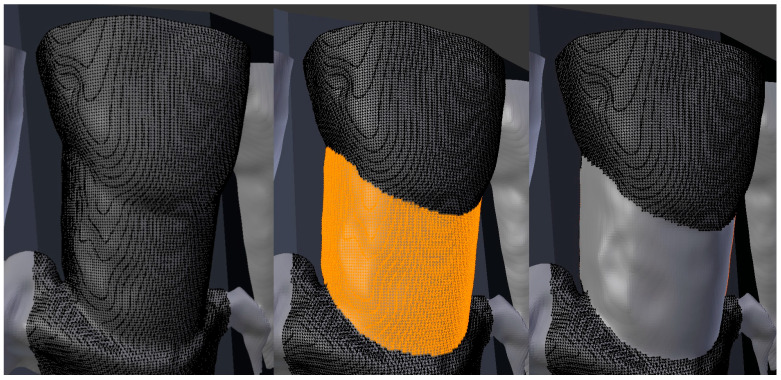
Exemplary marking of the defined vestibular defects.

**Figure 3:**
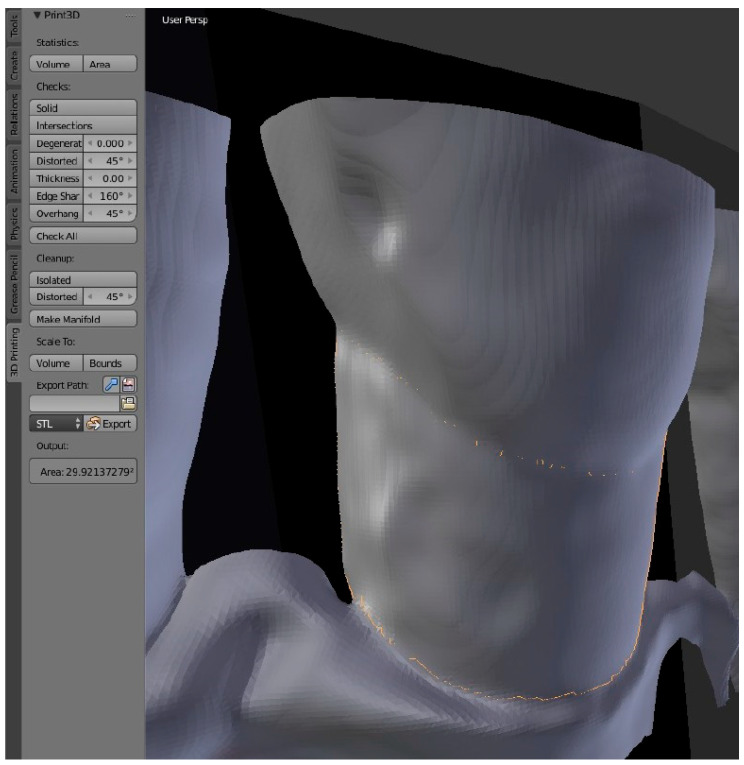
Exemplary output measurement of 3D graphics software for a vestibular defect.

Subsequently, defects were defined as the whole surface between the cementoenamel junction and residual bone displayed in front of the constructed segmentation surface (Figure 2). Marking of these surfaces was performed in the graphic software’s edit mode in three steps: (1) opening the edit mode, (2) marking the vertices of the defect visually, and (3) separating the selected vertices from the rest. The surfaces were measured in mm^2^ for all included teeth in all protocols via the software’s integrated 3D print tool and exported into a table (Figure 3).

### 2.2. Statistical Analysis

Statistical analysis was performed with IBM SPSS Statistics for Windows (Version 26.0, IBM Corp., Armonk, NY, USA) by an independent statistician. Intra-rater reliability for the operator of this study (J.B.) was determined to validate measurements. Therefore, following statistical consultation, the dehiscences of 32 teeth were subsequently measured two more times. The time intervals between measurements were six weeks each. Prior to analysis, the data were tested for normality. Statistical methods to analyze data were descriptive statistics, one-sample *t*-test (*p* < 0.05), and Bland–Altman plots (95% confidence interval, CI). The level of significance was set to *p* < 0.05.

## 3. Results

**Figure 4:**
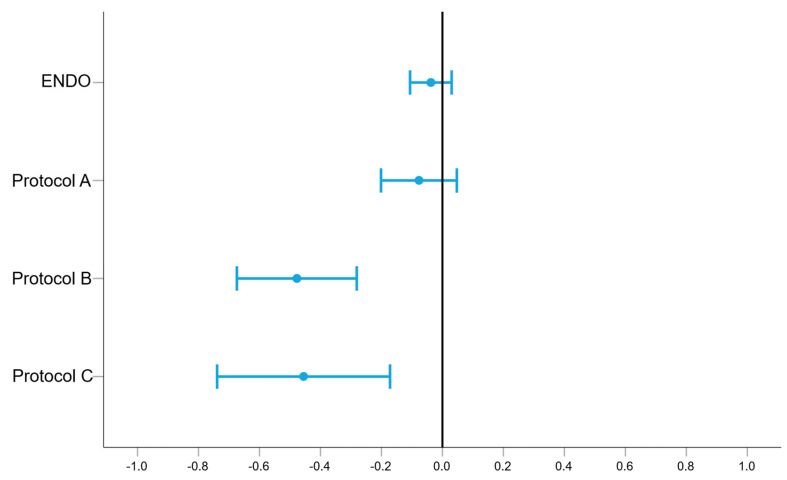
Forest plots visualizing the differences between CBCT protocols and reference datasets obtained with IOS. Abbreviation: CI (confidence interval), mm^2^ (square millimeter).

In total, CBCT scans of four skulls using four different settings were taken, and an IOS was utilized to obtain a reference dataset for each skull. Due to defects present in the skulls, the surfaces of 66 out of 80 teeth could be analyzed and compared. This resulted in an evaluation of 330 surfaces (5 × 66) within the 3D graphic software used, including the STL dataset generated by IOS. Results for all surface measurements are documented in detail in Table 2. The results of *t*-tests comparing the different CBCT protocols with the reference dataset obtained with IOS are visualized in forest plots in Figure 4. Respective *p*-values, mean, minimum, and maximum are shown in Table 3. While ENDO and protocol A confirmed the null hypothesis (*p* > 0.05), protocol B and C revealed significant differences (*p* < 0.05) compared to the reference. In general, the width of intervals and, thus, the documented random errors seemed to increase with larger voxel sizes of investigated CBCT protocols (Table 1 and Figure 4).

**Figure 5:**
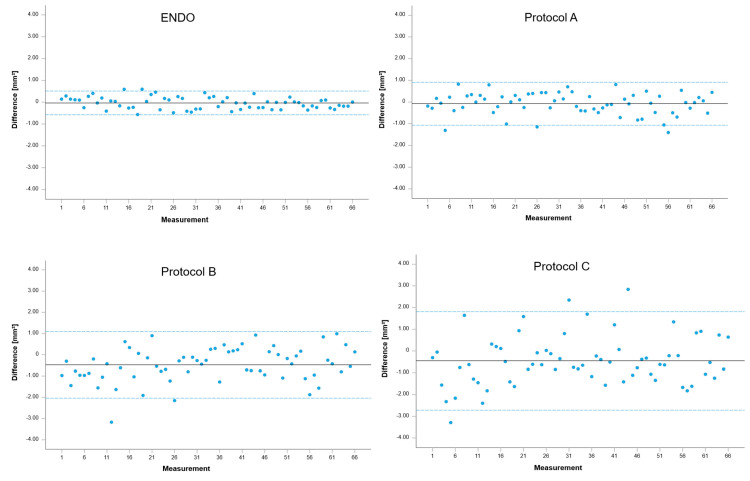
Bland–Altman plots visualizing and analyzing the agreement of measurements between CBCT protocols and reference datasets obtained with IOS. Abbreviation: mm^2^ (square millimeter).

Figure 5 shows Bland–Altman plots visualizing and analyzing the agreement of measurements between CBCT protocols and reference datasets. Respective Limits of Agreement (LoA) are documented in Table 4. Likewise, as documented for the width of intervals (Figure 4), LoA increased with the enlargement of voxels within the investigated CBCT protocols. The widened LoA thus confirms the significant differences already documented for protocols B and C.

**Figure 6:**
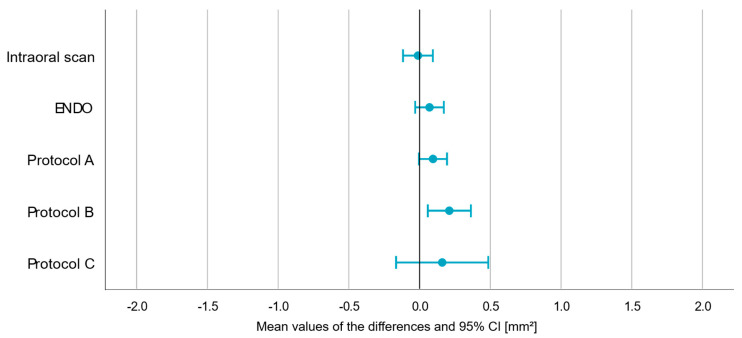
Forest plots visualizing the intra-rater reliability of all investigated datasets. Abbreviation: CI (confidence interval), mm^2^ (square millimeter).

Furthermore, the results of intra-rater reliability are documented in Figure 6, Table 5, and Table 6. Thereby, with a view to protocol B, the visualized forest plot shifts to the right, including its mean value (Figure 6). Without including zero and with an additionally documented *p* < 0.05, a human measurement error appears to have been documented here, in which the overall measured values are more positive than they should have been (Figure 6 and Table 5). Again, during repeated measurements of 32 datasets for the assessment of intra-rater reliability, LoA increased with the enlargement of the voxels used in the different CBCT protocols.

## 4. Discussion

In the present study, vestibular bony defects of single-rooted teeth were evaluated utilizing four different, commonly used CBCT protocols. Thereby, STL datasets obtained with an IOS served as references. While no statistically significant differences could be documented for ENDO and protocol A, protocols B and C revealed significant differences, which led to the working hypothesis having to be partially rejected. Furthermore, the examination of intra-rater reliability showed a statistically significant deviation regarding protocol B, which may indicate the possibility of individual measurement errors. Meanwhile, the largest LoA interval was documented for protocol C, suggesting greater dispersion of the measured data in the protocol with the largest voxel size and, therefore, the lowest resolution.

Human skulls are frequently used as a resource in studies evaluating human anatomy, CBCT dose, or in investigations involving different mAs protocols [37,38,39,40]. They can be particularly useful in enhancing clinical understanding, supporting education and training, and in the development or refinement of new methods [40,41]. Therefore, cadaver-based studies may also contribute to advancing modern implantology and reducing overall radiation exposure. However, as highlighted by Yeung et al., skulls may not provide optimal methodological quality or reflect clinical conditions accurately [42]. Although the additional use of soft tissue simulators would have been methodologically desirable, it was not permitted on the human donations provided. In contrast, other commonly utilized reference models, such as animal specimens or artificial phantom heads, are considered to possess even lower clinical relevance.

To evaluate the accuracy and, thus, applicability of different CBCT protocols, a corresponding reference is crucial. With advancements in digital technology, IOS offers a simple and cost-effective method for creating control groups. Thereby, the overall accuracy of IOS, including the specific device used in this study (CS 3600), has been scientifically validated in previous studies [43,44,45,46]. Nonetheless, it should be noted that the exported STL data from IOS represents a 3D surface composed of numerous small, interconnected triangles. Therefore, they do not fully reflect the actual physical reality. Furthermore, to the authors’ knowledge, there is currently no scientific consensus on the exact accuracy required for intraoral scanners. In a narrative review by Jennes et al. (2022), values ranging from 12.9 to 80.01 µm for trueness and from 42.9 to 86.0 µm for precision in full-arch dentition were reported [46]. While these low deviations are particularly relevant for the fabrication of dental prostheses, they can be considered negligible for the determination of vestibular bone defects in this study and the conclusions drawn therefrom.

For comparison, it was also necessary to export the CBCT datasets, originally stored in DICOM (Digital Imaging and Communications in Medicine) format, as STL files. Although the manufacturer-provided software, integrated with the CBCT system (Romexis), is designed for medical use, the authors have no further information regarding the accuracy of renderings and export of STL files. This issue represents not only a challenge for the present study but also a general problem in digitalization, as closed software systems provide limited transparency regarding the potential impact of various processing steps or software updates on the data output. However, Romexis has been previously compared with other programs in terms of accuracy, with no clinically significant differences [47,48,49]. Therefore, despite the lack of information regarding accuracy, the applied experimental setup appeared to provide a reliable basis for the comparison of the respective exported STL datasets.

Before selecting and evaluating the defect surfaces as outlined in the materials and methods section, additional considerations should be noted: IOS is restricted to capturing visible surfaces, whereas CBCT data encompasses additional information, such as bone pockets, that might not be detectable by IOS. To allow for a valid comparison, the defects in the CBCT mesh were marked at 90° angles relative to the defect surface, simulating a perspective that aligns with the capabilities of IOS. Additionally, a precise definition of the surface to be evaluated was essential for further analysis. For practical reasons, defect surfaces were defined as the space between the cementoenamel junction and residual bone displayed in front of the constructed boundary surface. Given that the present study does not investigate actual clinical data, employing this definitional approach for identifying defects streamlined the complex and time-consuming process of evaluating 330 surfaces while effectively demonstrating the underlying concept.

The 3D graphic software utilized in this study (Blender) is an open-source program with versatile functionality and 3D model editing capabilities, enabling necessary superimposition and data export. It was selected for its flexibility and efficiency in modifying 3D models, making it widely adopted in the dental field. Therefore, it is also used in other medical disciplines, such as surgical planning for rhinoplasty and the creation of training models for laryngoplasty [50,51]. Nevertheless, when interpreting the results, it is important to consider that the evaluation is based on small digital triangular elements, the accuracy of which represents an approximation of reality. Additionally, software-specific variations introduced by the manufacturer may again influence the outcomes.

The aim of this study was to analyze bone defects based on CBCT data and confirm its applicability within the workflow of immediate implant planning. Thereby, area-based measurements were assessed for a more comprehensive assessment of total bone loss. Although area measurements do not inherently indicate whether bone loss is vertical, horizontal, or combined, this distinction can be additionally determined during visual CBCT analysis. In comparison, linear (1D) measurements might display defects too simply and fail to distinguish between horizontal and vertical defects, often leading to an underestimation of defect size.

Protocols B and C revealed significant differences (*p* < 0.05) compared to reference datasets obtained by IOS. In general, the random error within this investigation seemed to increase with voxel size, which is represented by the *p*-values from *t*-tests. Additionally, forest plots seemed to visualize a systematic error in which the measured values of CBCT protocols B and C had shifted to the left. This could be attributed to the lower resolution of radiographs obtained with larger voxel sizes, resulting in blurred tissue interfaces. The reduced sharpness can affect both the post-processing and subsequent comparison of the respective defect surfaces. Consequently, protocol C, which utilized the largest voxel size, exhibited the widest LoA (−2.72–1.81 mm^2^). However, to the knowledge of the authors, no scientifically accepted standard or classification system for the evaluation of 3D bony defects is currently known. Present investigations have mainly focused on the accuracy of linear bone measurements on cross-sectional CBCT images [23]. Although it remains partially controversial, according to the 2023 ITI Consensus Statement, an intact or minimally damaged facial bone wall constitutes a selection criterion for immediate implant placement [52]. While no threshold values for the 3D size of defect areas were defined by ITI, a consensus report of group 3 of the XV European Workshop in Periodontology (2019) recommended that immediate implant placement should be avoided in extraction sites with severely damaged sockets (more than 50% loss of one or more walls) [53]. Therefore, based on the results of our study, inaccuracies of measurements of up to ±3 mm^2^ are unlikely to influence clinical decision-making regarding immediate implant placement, as such deviations in 3D defect size likely represent less than 50% of the total facial bone wall. Although the deviations observed in protocols B and C compared to the reference reached statistical significance, from the authors’ perspective, they are considered unlikely to be of clinical relevance given the measured values. Nonetheless, in complex cases where diagnostic uncertainty exists, the use of higher-resolution CBCT imaging is recommended.

Furthermore, the examination of intra-rater reliability showed a statistically significant deviation regarding protocol B (*p* < 0.05). The assessment of intra-rater reliability is primarily used for investigating potential human error. A similar rationale, as previously discussed, could be applied regarding voxel sizes, as larger voxel sizes might amplify the impact of human error. However, the absence of significant differences in protocol C contradicts this assumption. Additionally, the absence of inter-rater reliability analysis represents a limitation in assessing overall reproducibility. While our study focused on intra-rater consistency to establish baseline reliability, additional variability introduced by operator experience, training, or familiarity with the software could influence measurement accuracy. An inter-operator analysis could have provided further insights, though this was beyond the scope of the current investigation and may serve as an idea for future research. Finally, it should also be discussed as a limitation of this study that the analysis was conducted on a single CBCT device. Hardware-specific differences in voxel rendering can arise from variations in processing power, memory capacity, and manufacturer-specific algorithms. These factors may influence the accuracy, image quality, and processing speed of 3D reconstructions derived from voxel data. Therefore, differences in CBCT performance led to the development of quality control guidelines [54]. The results of this study should, therefore, not be uncritically extrapolated to all other devices. However, they may provide an indication of the applicability of ULD protocols for the assessment of bone defects and serve as a basis for further studies, such as device comparison analyses.

The findings of this study demonstrate that vestibular bony defects can be measured using ULD CBCT protocols. Although it revealed statistically significant differences in surface measurements between the different protocols, these differences seemed to remain clinically acceptable. Therefore, such protocols can be applied in clinical practice, adhering to the ALADAIP principle stated by DIMITRA. These findings could also be valuable for accurate virtual implant planning within immediate protocols while reducing radiation exposure and determining the possible need for additional hard or soft tissue augmentation procedures in advance. Thereby, preoperative knowledge of the anticipated clinical scenario can significantly influence the surgeon’s decision-making process. This might include the selection of implant installation timing, the selection and modification of additional required surgical procedures, and, if necessary, referral to a specialist [52].

## 5. Conclusions

This present study demonstrated that vestibular bony defects of single-rooted teeth can be reliably assessed using ULD CBCT protocols. While the two protocols showed statistically significant deviations in area measurements compared to the reference datasets, the extent of these deviations (defined threshold ±3 mm^2^) appears to be clinically negligible and unlikely to affect decision-making in immediate implant planning. The findings support the clinical applicability of ULD protocols in accordance with the ALADAIP principle, offering a reduction in radiation dose for diagnostic imaging. However, results should not be uncritically transferred to other CBCT devices, as hardware-specific differences may influence accuracy. Future studies, including inter-operator and inter-device comparisons, are recommended to further validate these outcomes and promote standardized, low-dose imaging protocols in implant dentistry. 

## Figures and Tables

**Table 1:** Examined CBCT protocols and their detailed settings.

Protocol Name	FOV (in mm)	Voltage (in kV)	Voxel (in µm)	Current (in mA)	Dose (in µSv)	Exposure Time (in s)
ENDO	40 × 50	90	75	12	80–120	15
A	50 × 50	90	75	9	20–40	5
B	100 × 60	90	150	7.1	22–32	5
C	100 × 100	90	200	7.1	44	4

Abbreviation: CBCT (cone-beam computed tomography), FOV (field of view), mm (millimeter), kV (kilovolts), µm (micrometer), µSv (micro-Sievert), s (seconds).

**Table 2:** Detailed results of all surface measurements.

**Skull 1**		FDI region	15	14	12	11	21	22	23	24	25	
	**Measured Surfaces in mm^2^**	IOS	20.25	37.44	34.45	20.17	24.90	31.68	43.09	20.42	21.93	
		ENDO	20.38	37.72	34.59	20.28	25.00	31.43	43.36	20.82	21.89	
		Protocol A	20.06	37.15	34.62	20.12	23.60	31.90	42.69	21.24	21.68	
		Protocol B	19.27	37.14	33.00	19.40	23.94	30.71	42.22	20.22	20.37	
		Protocol C	19.93	37.39	32.89	17.85	21.61	29.51	42.33	22.06	21.30	
		FDI region	45	44	43	42	41	31	32	33	34	35
	**Measured Surfaces in mm^2^**	IOS	22.26	11.43	30.94	19.61	17.43	19.14	22.35	24.97	7.51	18.15
		ENDO	22.45	11.02	31.00	19.65	17.27	19.73	22.08	24.73	6.94	18.74
		Protocol A	22.54	11.77	30.94	19.92	17.57	19.93	21.86	24.75	7.75	17.13
		Protocol B	21.21	11.00	27.77	17.98	16.81	19.76	22.69	23.93	7.57	16.24
		Protocol C	20.97	9.97	28.54	17.79	17.75	19.35	22.46	24.48	6.09	16.51
**Skull 2**		FDI region	14	13	12	11	21	22	23	24		
	**Measured Surfaces in mm^2^**	IOS	14.28	22.59	19.86	15.96	15.48	17.29	18.44	14.83		
		ENDO	14.31	22.94	20.32	15.60	15.65	17.39	17.95	15.09		
		Protocol A	14.28	22.89	19.96	15.70	15.85	17.69	17.29	15.27		
		Protocol B	14.13	23.49	19.32	15.18	14.79	16.06	16.29	14.54		
		Protocol C	15.21	24.18	19.01	15.34	15.39	16.66	18.46	14.71		
		FDI region	44	43	42	41	31	32	33	34		
	**Measured Surfaces in mm^2^**	IOS	10.42	15.69	16.59	13.13	16.55	19.29	25.82	11.49		
		ENDO	10.59	15.28	16.13	12.81	16.24	19.72	26.02	11.75		
		Protocol A	10.86	15.43	16.64	13.59	16.69	19.99	26.29	11.28		
		Protocol B	10.30	14.90	16.47	12.85	16.11	19.02	26.07	11.78		
		Protocol C	9.57	15.34	17.39	15.47	15.80	18.47	25.16	13.18		
**Skull 3**		FDI region	13	12	11	21	22	23	24			
	**Measured Surfaces in mm^2^**	IOS	13.71	11.25	11.70	20.90	10.10	20.04	14.46			
		ENDO	13.50	11.26	11.90	20.47	10.06	19.71	14.42			
		Protocol A	13.31	10.83	11.94	20.58	9.61	19.77	14.33			
		Protocol B	12.43	11.71	11.83	21.08	10.33	20.56	13.75			
		Protocol C	12.53	11.01	11.30	19.33	9.58	21.24	14.53			
		FDI region	45	44	43	42	41	31	32	33	34	35
	**Measured Surfaces in mm^2^**	IOS	12.82	26.22	15.18	7.72	15.04	26.40	6.89	30.78	22.41	9.07
		ENDO	12.59	26.61	14.92	7.47	15.06	26.06	6.88	30.43	22.40	9.30
		Protocol A	12.70	27.02	14.46	7.85	14.95	26.71	6.05	29.98	22.91	9.01
		Protocol B	12.07	27.15	14.42	6.77	15.18	26.83	6.89	29.69	22.23	8.63
		Protocol C	11.40	29.06	14.06	6.94	14.66	26.07	5.82	29.43	21.79	8.42
**Skull 4**		FDI region	15	14	13	11	21	22	23	24	25	
	**Measured Surfaces in mm^2^**	IOS	15.86	15.05	21.73	24.24	27.27	19.70	29.42	13.30	10.28	
		ENDO	15.87	15.03	21.56	23.88	27.10	19.45	29.49	13.40	10.02	
		Protocol A	15.38	15.32	20.67	22.84	26.77	19.00	29.95	13.28	10.00	
		Protocol B	15.80	15.21	20.61	22.37	26.32	18.13	30.26	13.04	9.86	
		Protocol C	15.64	16.39	21.51	22.57	25.44	18.08	30.25	14.20	9.22	
		FDI region	43	42	31	32	33					
	**Measured Surfaces in mm^2^**	IOS	16.56	13.71	12.68	15.37	44.31					
		ENDO	16.22	13.57	12.49	15.18	44.31					
		Protocol A	16.53	13.92	12.73	14.85	44.75					
		Protocol B	17.54	12.92	13.15	14.81	44.44					
		Protocol C	16.02	12.47	13.41	14.53	44.94					

Abbreviation: mm^2^ (square millimeter), FDI (Fédération Dentaire Internationale), IOS (intraoral scanner).

**Table 3:** Documented values regarding the differences between CBCT protocols and reference datasets obtained with IOS.

	*p*-Value	Mean (in mm^2^)	Min. (in mm^2^)	Max. (in mm^2^)
ENDO	0.271	−0.038	−0.106	0.030
A	0.220	−0.077	−0.201	0.047
B	0.000	−0.477	−0.674	−0.281
C	0.002	−0.455	−0.739	−0.172

Abbreviation: CBCT (cone-beam computed tomography), IOS (intraoral scanner), min. (minimum), max. (maximum), mm^2^ (square millimeter).

**Table 4:** Limits of Agreement (LoA) taken from Box–Altman plots within Figure 5 and respective *p*-values from *t*-tests considering Table 3.

	Limits of Agreement (in mm^2^)	*p*-Values (of *t*-Tests)
ENDO	−0.58–0.51	0.271
A	−1.07–0.91	0.220
B	−2.04–1.09	0.000
C	−2.72–1.81	0.002

Abbreviation: mm^2^ (square millimeter).

**Table 5:** Documented values regarding intra-rater reliability of all investigated datasets.

	*p*-Value	Mean (in mm^2^)	Min. (in mm^2^)	Max. (in mm^2^)
IOS	0.829	−0.011	−0.117	0.094
ENDO	0.165	0.070	−0.031	0.172
A	0.062	0.095	−0.005	0.194
B	0.008	0.211	0.058	0.363
C	0.324	0.160	−0.165	0.485

Abbreviation: IOS (intraoral scanner), min. (minimum), max. (maximum), mm^2^ (square millimeter).

**Table 6:** Limits of Agreement and respective *p*-values considering intrarater reliability.

	Limits of Agreement (in mm^2^)	*p*-Values (of *t*-Tests)
IOS	−0.58–0.56	0.829
ENDO	−0.48–0.62	0.165
A	−0.45–0.64	0.062
B	−0.62–1.04	0.008
C	−1.61–1.93	0.324

Abbreviation: IOS (intraoral scanner), mm^2^ (square millimeter).

## Data Availability

The data presented in this study are available on request from the corresponding author.
